# Transcriptomic characterisation of acute myeloid leukemia cell lines bearing the same t(9;11) driver mutation reveals different molecular signatures

**DOI:** 10.1186/s12864-025-11415-1

**Published:** 2025-03-25

**Authors:** Elise Georges, William Ho, Miren Urrutia Iturritza, Lel Eory, Kamila Malysz, Ulduz Sobhiafshar, Alan L. Archibald, Daniel J. Macqueen, Barbara Shih, David Garrick, Douglas Vernimmen

**Affiliations:** 1 The Roslin Institute and Royal (Dick) School of Veterinary Studies, University of Edinburgh, Easter Bush, Midlothian, EH25 9RG UK; 2 Present Address: Division of Biomedical and Life Sciences, Faculty of Health and Medicine, Lancaster University, Lancaster, UK; 3 INSERM UMR 1342, Institut de Recherche Saint Louis, Université Paris Cité, Paris, 75010 France

**Keywords:** Leukemia, Acute myeloid leukemia, Comparative transcriptomics, Zinc finger (ZNF) transcriptional repressors

## Abstract

**Background:**

Acute myeloid leukemia (AML) is the most common type of acute leukemia, accounting for 20% of cases in children and adolescents. Genome-wide studies have identified genes that are commonly mutated in AML, including many epigenetic regulators involved in either DNA methylation (*DNMT3A*, *TET2*, *IDH1/2*) or histone post-translational modifications (*ASXL1*, *EZH2*, *MLL1*). Several cell lines derived from AML patients are widely used in cancer research. Whether important differences in these cell lines exist remains poorly characterised.

**Results:**

Here, we used RNA sequencing (RNA-Seq) to contrast the transcriptome of four commonly used AML-derived cell lines: THP-1, NOMO-1, MOLM-13 bearing the common initiating t(9;11) translocation, and MV4.11 bearing the t(4;11) translocation. Gene set enrichment analyses and comparison of key transcription and epigenetic regulator genes revealed important differences in the transcriptome, distinguishing these AML models. Among these, we found striking differences in the expression of clusters of genes located on chromosome 19 encoding Zinc Finger (ZNF) transcriptional repressors. Low expression of many ZNF genes within these clusters is associated with poor survival in AML patients.

**Conclusion:**

The present study offers a valuable resource by providing a detailed comparative characterisation of the transcriptome of cell lines within the same AML subtype used as models for leukemia research.

**Supplementary Information:**

The online version contains supplementary material available at 10.1186/s12864-025-11415-1.

## Introduction

Leukemia results from the accumulation of mutations in oncogenes (gain-of-function) and/or tumour suppressor genes (TSG) (loss-of-function), giving rise to an imbalance between proliferation/self-renewal and differentiation in the hematopoietic stem and progenitor cell (HSPC) pool [1]. In acute myeloid leukemia (AML), initiating mutations (such as the commonly occurring AF9-MLL translocation) dysregulate stem cell fate decisions in HSPCs, generating pre-leukemic stem cells (pre-LSCs) [2]. Under permissive conditions, pre-LSCs acquire cooperating mutations which generate leukemic stem cells (LSCs) that drive leukemogenesis and are difficult to eradicate using currently available treatments [3, 4].

The primary and secondary genetic targets that drive the emergence of LSC and eventually leukemogenesis can be broadly classified as either conferring proliferative and survival advantages (e.g. *FLT3*, *KIT*, *RASs*), altering differentiation and apoptosis (e.g. *PML-RARA*, *RUNX1*, *MLL1*), and a third important category of mutations which leads to an alteration of cellular epigenetic state [5, 6]. Many of the mutations in this last group (including *DNMT3*, *TET2* and *IDH1/2*) affect the DNA methylation machinery, disrupting normal DNA methylation in LSC. Other mutations (including *ASXL1*, *EZH2 and MLL1*) affect proteins involved in post-translational modifications of histones, which are important for transcriptional regulation, DNA repair, regulation of the cell cycle and differentiation [7]. Epigenetic pathways are of particular interest from a therapeutic perspective, due to the inherent plasticity of epigenetic modification patterns and because many epigenetic regulators can be targeted by drugs [8–11], some of which are currently approved for clinical applications [12].

The choice of cell line to be used as an experimental model to study leukemia in the laboratory is a complex multi-factorial decision, which has important ramifications for the results observed and their interpretation. The most important considerations include the disease subtype represented by a cell line (most frequently indicated by the French-American-British (FAB) classification system) as well as the different driver genetic events (chromosomal translocations or mutations) [13]. However, immortalised cells also bear multiple secondary genetic mutations that exert widespread quantitative and qualitative alterations on the molecular characteristics of the cell, including the transcriptome. The influence of this complexity on cellular phenotype is frequently overlooked in the interpretation of experimental findings.

Fundamental research on AML is often performed in one of several well-established cell line models. However, many of these commonly used cell lines remain incompletely characterised, and, in particular, direct molecular comparisons of these different models are lacking. Here, we have used RNA sequencing (RNA-Seq) to compare the transcriptome of three commonly used AML cell lines bearing a common driver event, the t(9;11) translocation (THP-1, NOMO-1, MOLM-13) together with another AML cell line (MV4.11) bearing a different translocation, t(4:11). Pairwise comparisons between all cell lines revealed that pathways related to the regulation of gene transcription were among the most significantly different between the cell lines. More specifically, we found alteration of the p53 pathway, the HOX gene clusters, key hematopoietic transcription factors and epigenetic regulators, all relevant to AML. We also observed striking differences in the expression of genes encoding C2H2 Zinc Finger (ZNF) transcriptional repressors clustered on chromosome 19 (Chr19) which have prognostic significance in AML patients. This study provides a transcriptomic resource together with additional insights that will improve the choice of AML cell lines as models for pre-clinical research such as drug screens.

## Results and discussion

### Transcriptomic characterisation of AML cell line models

All AML cell lines used in this study belong to the same subtype (M5) classification of the FAB system [13]. THP-1, MOLM-13 and NOMO-1 are characterised by a t(9;11) translocation (p22;q23 involving *MLLT3-KMT2A* fusion genes) [14, 15] common in AML, whereas MV4.11 has a t(4;11) translocation (q21;q23 involving *AFF1-KMT2A* fusion genes) [16], which is more common in acute lymphoblastic leukemia (ALL) [17], but with myeloid features [18, 19]. Induction of t(9;11) or t(4;11) is sufficient to promote myeloid [20] and lymphoblastic [21] leukemia respectively. Of the three t(9;11) cell lines, THP-1 was derived from a 1-year old male [22]; NOMO-1 from a 31-year old female [23]; and MOLM-13 from a 20-year old male [15]. MV4.11 was derived from a 10-year old male [16].

**Fig. 1:**
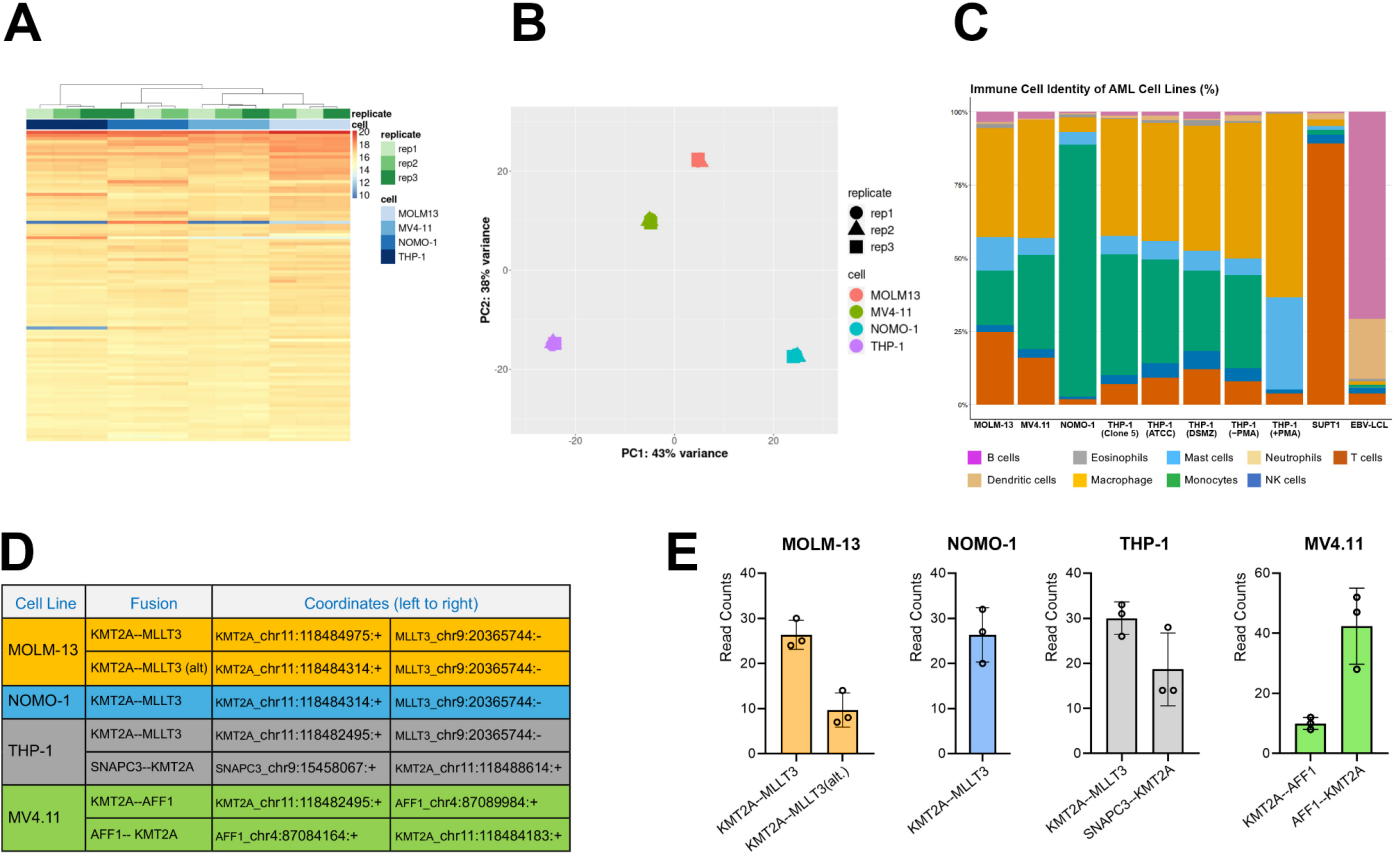
Characterisation of AML cell lines’ identity. (**A**) Sample clustering based on expression distances after a variance stabilizing transformation of read counts. (**B**) Principal component analysis (PCA) of the AML cell line dataset. (**C**) Characterisation of AML cell line identity. Inferred phenotypic profiles of the four AML cell lines were generated with CIBERSORTX. Published RNA-Seq data from THP-1 obtained from ATCC and DSMZ cell line repositories were used as a comparison with our own THP-1 dataset (Clone 5). The phenotypic characteristic of THP-1 is completely altered when chemically induced to differentiate into macrophages in culture (THP-1 control vs. THP-1 + PMA). Published RNA-Seq data from SUPT1 and EBV cell lines is included as comparisons for T-cell and B-cell profiles, respectively. (**D**) Genomic coordinates of fusion transcripts spanning the primary breakpoints detected in the cell lines. (**E**) Quantification of junction reads for the MLL fusion transcripts. The counts shown indicate the number of RNA-Seq fragments containing a split read at the site of the fusion junction

To compare these cell lines, we performed RNA-Seq using deep sequencing (> 120 M paired-end reads per sample). Surprisingly, clustering and principal component analysis (PCA) revealed the closest concordance in transcriptomic profile between MV4.11 (t(4;11)) and MOLM-13 (t(9;11)), with the two other t(9;11) cell lines being more distally clustered (Fig. 1A and B) (discussed further below). Comparative analysis of cell-type characteristics using CIBERSORTX [24] revealed that NOMO-1 exhibited a predominantly monocytic transcriptome, with the other three cell lines exhibiting stronger macrophage characteristics (Fig. 1C). As reported in previous studies [25, 26], our RNA-Seq datasets revealed the presence of multiple distinct fusion transcripts involving the primary breakpoint in MOLM-13, MV4.11 and THP-1 cell lines (Fig. 1D, E). Specifically, in MOLM-13 two different break points were observed within the *KMT2A* locus, leading to 2 different chimeric transcripts of *KMT2A-MLLT3*. In the case of MV4.11, reciprocal fusion *KMT2A-AFF1* transcripts were detected (Fig. 1D, E). In THP-1, fusion transcripts were detected between *KMT2A* and two different partners on chromosome 9, *MLLT3* and *SNAPC3*. A full analysis of all fusion transcripts detected in each cell line is also provided in Supplementary Tables 1, 2, 3, and 4.

**Fig. 2:**
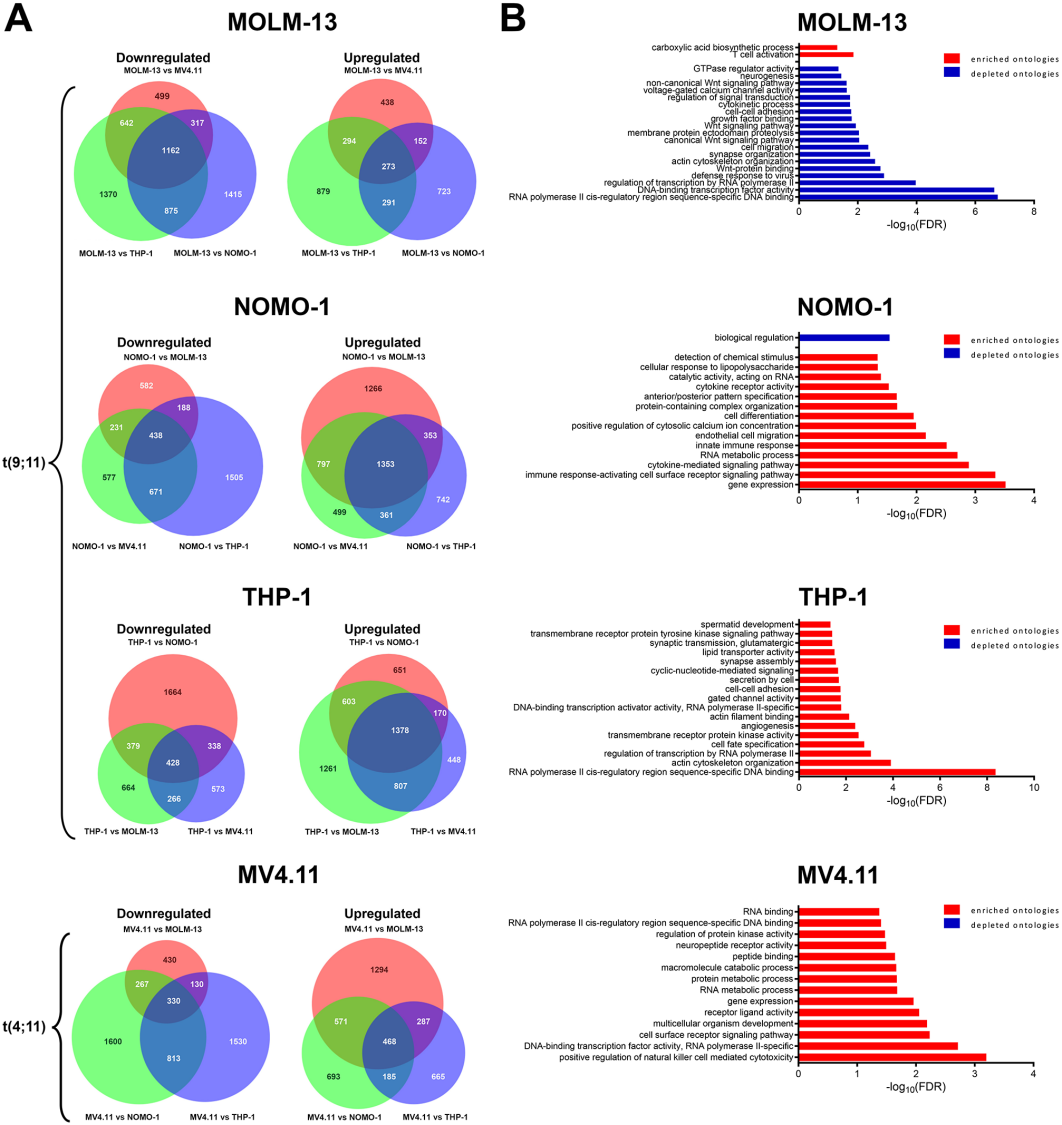
Differential expression analysis of AML cell line models. (**A**) Venn diagrams representing genes down- or up- regulated (cutoffs fold change > 2.5 and p_adj_ < 0.01) in the indicated cell line, relative to each of the three other cell lines. (**B**) The overlap of the gene sets (dark blue) in (**A**) was used in the GO enrichment analyses, where bar charts indicate GO terms significantly enriched (red) or depleted (blue) in each cell line (FDR-corrected *p* < 0.05)

To further explore differences between the transcriptomes, we carried out pairwise comparisons between all cell lines (Fig. 2, Supplementary Table 5). These analyses revealed widescale changes in gene expression, with between 3,777 and 5,786 differentially expressed genes (DEG) (cut-offs of fold change > 2.5 and p_adj_ < 0.01) observed between any cell line pair. For each cell line, we identified the genes specifically up- or down- regulated relative to all other three lines (Fig. 2A, Supplementary Table 5). Gene ontology (GO) enrichment tests revealed alteration of distinct biological processes and pathways (Fig. 2B). Specifically, NOMO-1 showed relative enrichment of signatures associated with cytokine and innate immune signalling pathways, MV4.11 of pathways associated with immune (Natural Killer) cell activation, signalling and protein kinase regulation, and THP-1 of pathways associated with regulation of the actin cytoskeleton (Fig. 2B). In contrast, MOLM-13 showed few enriched ontologies, but relative depletion of signalling pathways (particularly Wnt-signalling), and signatures of cell migration and adhesion. Notably, pathways related to transcription factor binding, RNA polymerase II activity and gene expression were among the most significantly altered pathways specific to each cell line, suggesting that alterations in transcriptional regulation are a primary determinant of the different characteristics of these cell lines, regardless of the translocation status.

### Differential expression of transcription factors and epigenetic regulators

Following the above observation, we focused further on differences in key transcription factors (TF) and epigenetic regulators which have been strongly implicated in the aetiology of AML. The members of the homeobox (*HOX*) family of TF are key regulators of normal hematopoiesis [27–29] and frequently dysregulated (mostly upregulated) in AML, where they have been associated as prognostic factors [30]. Focused analysis revealed striking differences between these AML cell lines in expression of the *HOX* genes (Supplementary Fig. 1). While most genes of the *HOXB* cluster are more prominently expressed in MV4.11 (t(4;11)) (Supplementary Fig. 1A, B), the *HOXA* cluster appears to be separated into two distinct regulatory subdomains, with the 5’ subdomain (HOXA*10*, *11* and *13*) also strongest in MV4.11, while the 3’ subdomain (*HOXA1*-*9*) is most prominently activated in NOMO-1 (t(9;11)) (Supplementary Fig. 1A, C). Interestingly, the 3’ and 5’ subdomains of the *HOXA* cluster have been shown to be subject to distinct epigenetic regulation and display different expression profiles during monocytic differentiation [31]. *HOXA9* and its cofactor *MEIS1*, have previously been identified as key target genes of leukemogenic MLL fusion proteins [2, 32–34] and enforced coexpression of both genes leads to rapid development of AML [35]. Of interest, we observed lowest expression of *HOXA9* and no expression of *MEIS1* in THP-1 (Supplementary Fig. 1D). The depletion of these key mediators of MLL activity in THP-1 is consistent with previous results indicating that THP-1 was less sensitive to depletion of MLL than MOLM-13, NOMO-1 and MV4.11 [33]. Interestingly, it has been previously shown that expression of *MEIS1* varies between THP-1 cells accessed from different biorepository [36].

**Fig. 3:**
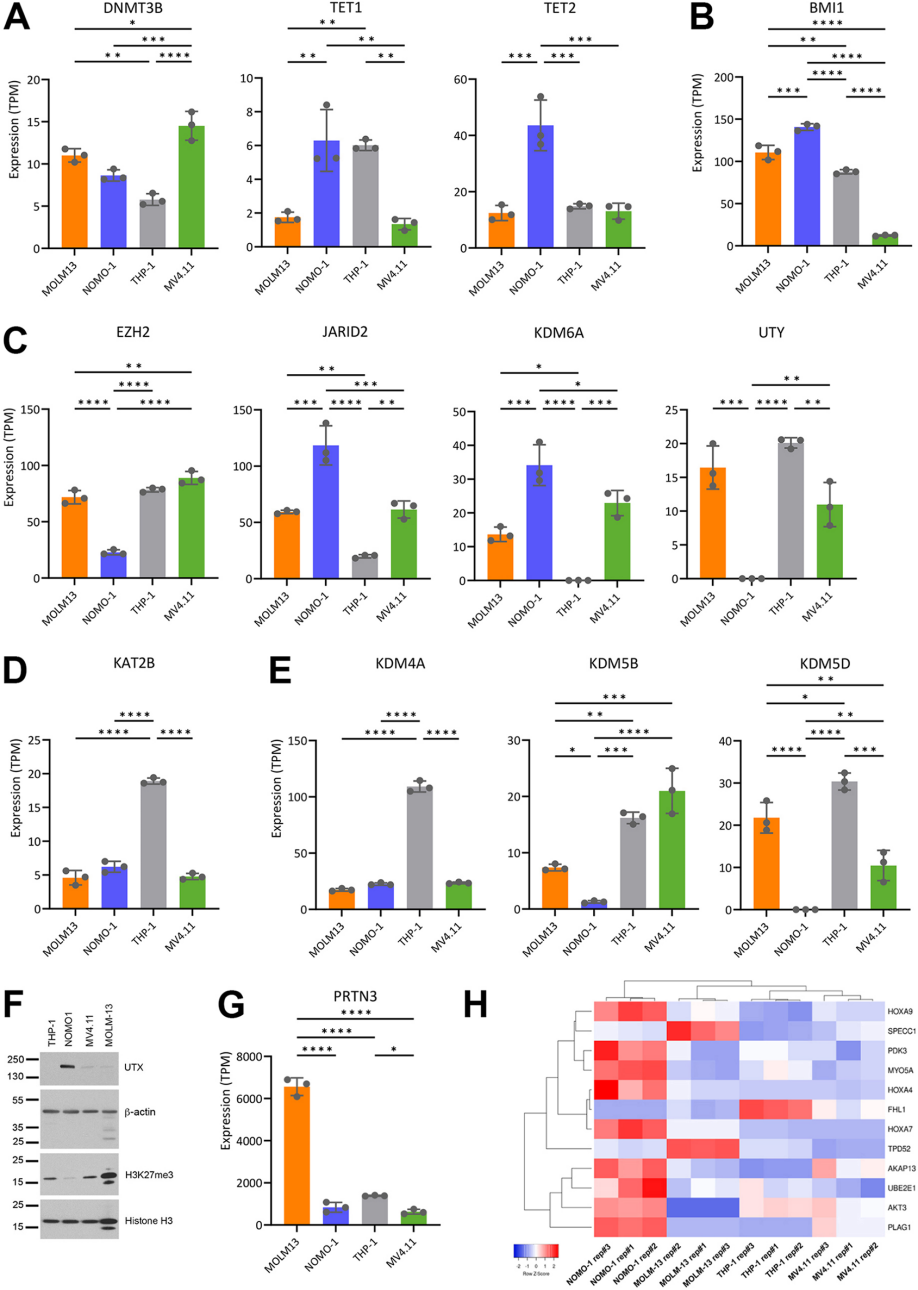
Differential expression of epigenetic regulators in AML cell lines. Bar charts showing expression (transcripts per million, TPM) in RNA-Seq datasets of the AML cell lines for the following genes: (**A**) Regulators of DNA methylation are represented by DNA methyltransferase *DNMT3B* and demethylases *TET1 and TET2*. (**B**) *BMI1* and (**C**) *EZH2* (H3K27 methyltransferase) and *JARID2* (DNA binding protein) are core components of polycomb repressive complexes 1 (PRC1) and 2 (PRC2) respectively, and *KDM6A*, *KDM6B*, *UTY* are H3K27 demethylases. (**D**) Lysine acetyl transferase *KAT2B* (**E**) Lysine demethylases (*KDM4A*, *5B*, *and 5D*). (**F**) Western blot analysis of UTX and H3K27me3 levels in AML cell lines. Both β-actin and Histone H3 were used as loading control. Recurrent protein degradation is notable in MOLM-13 cell line. (**G**) Bar charts showing expression of *PRTN3* (**H**) Clustered heatmap representation of genes previously shown to be repressed by PcG in AML (scaling applied to rows)

Our analysis also indicated differences between the cell lines in the expression of several epigenetic regulators known to be implicated in myeloid malignancies, including regulators of DNA methylation (Fig. 3A), components of the polycomb repressive complexes (PRC1, Fig. 3B and PRC2, Fig. 3C), and other histone modifying enzymes (lysine acetyl transferase *KAT2B*, Fig. 3D, lysine demethylases *KDM4A*, *KDM5B* and *KDM5D*, Fig. 3E). Other epigenetic regulator genes which can harbour driver mutations in AML (*DNMT3A*, *ASXL1*) [37–39] were expressed at similar levels in all four cell lines (Supplementary Table 6). Dysregulation of the Polycomb-mediated histone modification H3K27me3, including loss-of-function of the H3K27 methyltransferase EZH2 [40–43] or demethylase *KDM6A*/UTX [44–50] are common features of myeloid malignancies. As reported previously [51–53], western blot and whole genome sequencing (WGS) analysis confirmed that THP-1 lacks expression of UTX due to partial deletion of the single *KDM6A* allele (X-linked) in this cell line (Fig. 3C, Supplementary Fig. 2). Western blot analysis also revealed a striking depletion of the total levels of H3K27me3 in NOMO-1 (Fig. 3F), which is associated with both lower expression of EZH2 (Fig. 3C) as well as high expression of *KDM6A* gene (Fig. 3C) and its protein product UTX (Fig. 3F). Consistent with this observation, NOMO-1 also showed elevated expression of key genes which have been shown to be repressed by *EZH2* in AML (Fig. 3H), including target genes implicated in chemotherapy resistance, relapse, and poor prognosis [42]. Altogether, these findings suggest important differences in epigenetic regulation between the compared AML models.

Of note, the western blot analyses also suggested higher levels of protein degradation in MOLM-13 (Fig. 3F), which also displayed very high expression of *PRTN3* encoding Proteinase 3 (PR3, myeoblastin, Fig. 3G), a serine protease localized on the cellular membrane of polymorphonuclear neutrophils and identified as a leukemia associated antigen [54]. These observations suggest that MOLM-13 may not be the AML model of choice for carrying out proteomic studies.

We also identified significant differences in the expression of key transcriptional regulators of hematopoiesis such as *GATA2*, *TAL2* and *NF-E2* (Supplementary Fig. 3), all hallmarks of hematological malignancies [28, 55–79]. Other key regulators of hematopoiesis known to be involved in AML such as *JUND*, *ZEB1*, *IRF*, *C/EBP* or Forkhead Box (*FOX*) families [68, 72–74, 76–96] also present notable differences in expression between the four cell lines (Supplementary Fig. 3). Striking differences in expression were also observed for other important regulators of hematopoiesis, including *CITED4* and *NFYC* [97–99] (both strongly expressed in THP-1) (Supplementary Fig. 3). Of particular note, *TP53* was expressed only in MV4.11 (t(4;11)) and MOLM-13 (t(9;11)), the two cell lines whose transcriptomes clustered most closely, despite bearing different primary translocations (Fig. 1A, B). Interestingly, ontology analysis of genes which are upregulated in MOLM-13 or MV4.11 relative to THP-1 and NOMO-1, revealed a striking enrichment of p53 related signatures (Supplementary Fig. 4A, B, C & D), suggesting that common activation of the p53 signalling pathway may be one important factor contributing to the transcriptional similarity of these two cell lines. In addition to the deletion of one allele of *TP53* which has previously been described in THP-1 [100], WGS of this cell line also confirmed a 26 bp frameshift deletion in exon 5 of the other *TP53* allele [101] (Supplementary Fig. 4E), likely contributing to nonsense-mediated mRNA decay [102]. A frameshift mutation (p.C242fs*5) has also been previously reported in one TP53 allele in NOMO-1 [100].

**Fig. 4:**
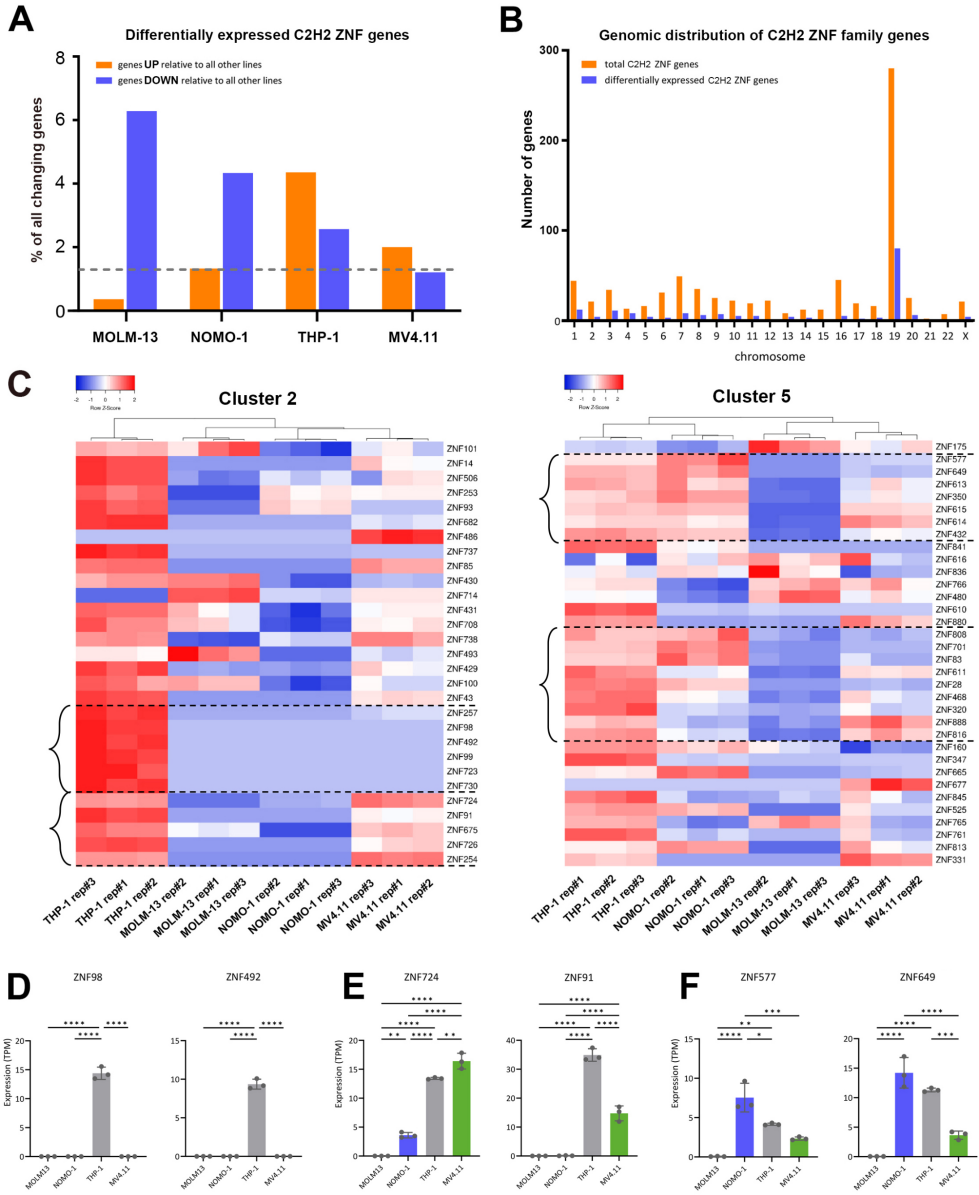
Differential expression of C2H2 Zinc Finger family transcription factors in AML cell lines. (**A**) Bar charts indicating the percentage of genes specifically up- or down- regulated in each cell line which are members of the C2H2 ZNF family. The dashed line indicates the overall percentage of this family across the genome (**B**) Bar charts indicating the number of total and differentially expressed C2H2 ZNF genes on each chromosome. Differentially expressed ZNFs are those that showed differential expression (in the same direction) between one cell line and all other three cell lines in pairwise comparisons as shown in Fig. 2. (**C**) Heatmap representation of the expression of ZNF genes within cluster 2 (left) and cluster 5 (right) on chromosome 19 (scaled within rows). Hierarchical clustering was applied only to columns (samples) and ZNF genes are shown according to their physical position within the gene cluster. Brackets indicate sub-clusters of physically-proximal genes showing co-ordinated expression profiles across the cell lines as described in the text. (**D**-**F**) Bar charts showing expression (TPM) of the indicated ZNF genes from different subclusters as described in the text

**Fig. 5:**
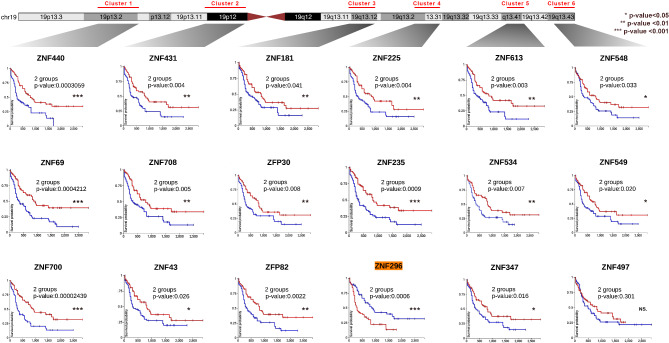
Survival analysis of ZNF genes on chromosome 19. (**A**) Map of chromosome 19 indicating the location of the 6 main clusters of ZNF genes. (**B**) Kaplan-Meier analysis of overall survival in patients of the LAML cohort (*n* = 132) with low (< median, blue) or high (> median, red) expression of selected ZNF genes within each cluster. The gene highlighted in orange (ZNF296) has the opposite behaviour to that observed for all the other genes

One striking observation from our data was that among genes that are differentially expressed between cell lines, there was a strong enrichment for members of the C2H2 Zinc finger (ZNF) family (HGNC Group ID 28) (Fig. 4A, Supplementary Table 6). Compared to their overall abundance in the human genome (1.3% of annotated genes), the *C2H2 ZNF* family was particularly enriched among the set of genes that were specifically depleted in MOLM-13 (73 out of 1162 genes, 6.3%) and NOMO-1 (19 out of 438 genes, 4.3%) and among the set of genes that were specifically increased in THP-1 (60 out of 1378 genes; 4.4%). Approximately half of the *C2H2 ZNF* family contain a KRAB (Krüppel-associated box) transcriptional repressor domain [103, 104]. Members of the C2H2 ZNF family are predominantly localised on chromosome 19 (Fig. 4B), where they are organised within 6 main clusters [105] (Supplementary Fig. 5). Interestingly, we observed striking differences between the cell lines in expression of these individual clusters, including strong expression of cluster 2 in THP-1 and generally poor expression of cluster 5 in MOLM-13 (Fig. 4C and Supplementary Fig. 6). We also observed several clear examples of physically proximal subclusters of ZNF genes within the primary clusters which showed correlating expression profiles, suggesting co-ordinated local regulation of proximal genes, consistent with observations in other cell types [92]. For example, a sub-cluster of genes within cluster 2 (*ZNF257* to *ZNF730*) showed highly specific activation in THP-1 (Fig. 4C, D), while an adjacent subcluster (*ZNF724* to *ZNF254*) was elevated in both THP-1 and MV4.11 (Fig. 4C, E). Within cluster 5, two subclusters (*ZNF577* to *ZNF432* and *ZNF808* to *ZNF816*) with striking depletion in MOLM-13 (Fig. 4C, F) are separated by a distinct subcluster of four genes (*ZNF616* to *ZNF480*) which are highly expressed in this cell line (Fig. 4C). To explore further, we analysed the prognostic significance of expression of these chr19 ZNF genes in patients of The Cancer Genome Atlas (TCGA) Acute Myeloid Leukemia cohort [44]. Interestingly, we observed correlations between expression of many of these ZNF proteins and prognostic outcome, with low expression most frequently associated with poor overall survival (Fig. 5, Supplementary Table 7).

One important role of these KRAB-containing ZNF proteins is the maintenance of heterochromatin and transcriptional repression at genomic retroelements [105, 106]. Expression of these genes has been associated with pro- [106] or anti- [107] tumorigenic influences in different malignancies, suggesting disease-specific effects. As well as their DNA binding-dependent function, a recent study showed that many members of the C2H2 ZNF family also bind directly to RNA, and can regulate post-transcriptional processes of splicing, polyadenylation and m^6^A modification, significantly expanding our understanding of the regulatory importance of this protein family [104]. Although these ZNF clusters have not previously been implicated in AML, our observations suggest that differential expression of these ZNF proteins on chr19 could exert important functional effects in patients and in the cell line models studied here, and that the role of these ZNF in AML is worthy of further study and functional characterisation.

## Conclusion

While studying the complex biological processes involved in leukemia, it is of great importance to define the most suitable cell line model required for a specific study. The detailed transcriptomic profiling of four commonly used AML cell lines reported here has revealed important differences in the expression of genes encoding TFs and epigenetic regulators, several of which have previously been implicated in the aetiology of the disease. Importantly, most differences in expression of these factors were not overtly correlated with the primary cytogenetic translocation present in each cell line demonstrating the importance of thorough molecular characterisation of these cellular models.

## Materials and methods

### Cell lines

Cells lines were kindly donated by David Hume (THP-1, clone 5) and Kamil Kranc (NOMO-1, MOLM-13, MV4.11). The THP-1 subclone (clone 5) used in this study was previously selected for its greater ability to differentiate into macrophages [108]. All were cultured in RPMI 1640 with the same batch of endotoxin free FBS (10%) (Gibco Lot #08F7582K). All cells were tested for mycoplasma using the Venor™ GeM Mycoplasma Detection Kit (MP0025– Sigma-Aldrich).

### RNA extraction

Total RNA was extracted with Tri Reagent per the manufacturer’s instructions (AM9738– Thermo Fisher), followed by TURBO DNase treatment (AM2238– Thermo Fisher). RNA quality was assessed by Agilent Tapestation 2200 with all samples achieving a RIN > 9.5.

### RNA-Seq library Preparation and sequencing

The Illumina TruSeq Stranded mRNA kit was used for RNA-Seq library preparation, with one round of RiboZero Gold for ribo-depletion. Preparation of libraries was carried out in a single batch. Pooled libraries (one full lane per cell line) were sequenced together on a Illumina HiSeq 4000 instrument at Edinburgh Genomics (http://genomics.ed.ac.uk/*)* generating 2 × 150 bp paired-end reads. Read counts are shown in Supplementary Table 8.

### Bioinformatics analysis

RNA-Seq data was aligned to the reference human genome (GRCh38) using STAR (v 2.7.1a) [109] allowing a maximum of 20 multimappers per read. Mapping rates were very high in all cases, above 90% (Supplementary Table 8). Aligned RNA-Seq data was analysed using the immune cell deconvolution software CIBERSORTX [24] running on relative mode with 1,000 permutations, using the LM22 signature matrix (microarray profile of 22 defined human immune cell types) and subset to only display monocyte, M0, M1 and M2 macrophages. Quantification of fusion transcripts was carried out using STARFusion (v1.10.0). For differential expression analysis, gene level read counts were obtained using featureCounts (v. 1.6.3) [110]. Following removal of genes poorly expressed in all samples (maximum mapped read count < 20), and genes on the Y chromosome, genes which were differentially expressed between pairs of cell lines (fold change > 2.5 and p_adj_ < 0.01) were determined using the DESeq2 package with Median Ratio (MR) normalisation [111] in R [112]. Overlaps and Venn diagrams of up- or down- regulated genes in a single cell line compared to each of the other three cell lines were carried out using Biovenn [113]. Ontology analysis was performed using the Gene Ontology Resource tool (release 17.6.24) [114] with the GO Biological Process_Slim and GO Molecular Function_Slim collections [115] after applying a cutoff for enrichment or depletion of FDR q < 0.05. GO terms within the same hierarchy were reduced to retain only the most specialised “child” term. Ontology analysis in Supplementary Fig. 4 was performed using the “Compute Overlaps” tool of MSigDB (https://www.gsea-msigdb.org/gsea/msigdb/index.jsp), with the Ontology Gene Sets (C5), applying a cutoff for enrichment of FDR q < 0.05. Heatmaps were established from MRN-normalised expression values using Heatmaper [116] with scaling applied to rows (genes). Where shown, hierarchical dendograms indicate the outcome of complete linkage clustering using the Euclidean measure of distance. Gene expression bar charts display the mean ± SD of each distribution and were analysed by ordinary one-way ANOVA with Bonferroni’s correction for multiple testing using GraphPad Prism (v10.3.1). Only significant pairwise comparisons are indicated (* *p* < 0.05, ** *p* < 0.01, *** *p* < 0.001, **** *p* < 0.0001).

### Kaplan-meier survival analysis

The patient survival and expression data (FPKM-UQ) were extracted from TCGA Acute Myeloid Leukemia (LAML) [44] via Xena browser [117]. For each gene, patients were segregated into groups of high (> median, *n*≅66) and low (< median, *n*≅66) expressors and Kaplan-Meier survival analysis was carried out using GraphPad Prism 10.3.1. The Log-rank test p value is indicated.

### Whole genome sequencing

The genomic DNA of all AML cell lines was obtained using phenol/chloroform extraction. The genomic DNA was randomly sheared into short fragments. The obtained fragments were end repaired, A-tailed, and further ligated with Illumina adapters. The fragments with adapters were size-selected, PCR amplified and purified. The library was checked with Qubit and real-time PCR for quantification and Bioanalyzer to determine size distribution. Next Generation sequencing libraries were prepared using Illumina SeqLab specific TruSeq PCR-Free High Throughput library preparation kits. Sequencing was performed using NovaSeq X Plus (PE150) instrument by NOVOGENE at 25x coverage.

### Western blot

Cells were resuspended in 1x PBS and lysed with 1x pre-heated PBS with SDS 2% (1% final). The cells were then boiled at 95 °C for 5 min, with agitation every minute. Lysates were pipetted up and down or sonicated to reduce viscosity, if necessary, then diluted 1:1 in 2x loading (SB) buffer (130mM Tris pH 6.8, 5% SDS, 20% Glycerol, 10% β-Mercaptoethanol, 0.01% Bromophenol Blue). Ten µg of cell lysates were separated on SDS-PAGE gradient (NuPAGE Bis-Tris 4–12%). Antibodies used were Histone H3 (Abcam 1791), UTX (Cell Signaling Technology #33510), H3K27me3 by Millipore (07-449) and β-actin-HRP (Sigma, A3854).

## Electronic supplementary material

Below is the link to the electronic supplementary material.

Supplementary Material 1

Supplementary Material 2

Supplementary Material 3

Supplementary Material 4

Supplementary Material 5

Supplementary Material 6

Supplementary Material 7

Supplementary Material 8

Supplementary Material 9

Supplementary Material 10

Supplementary Material 11

Supplementary Material 12

Supplementary Material 13

Supplementary Material 14

## Data Availability

THP-1 RNA-Seq data sets from ATCC and DSMZ cell repositories were obtained through the Gene Expression Omnibus (GEO) GSE130985 [36]. RNA-Seq data set for control and PMA treated THP-1 were obtained from BioProject PRJNA449980 [118], SUPT1 from BioProject PRJNA449980 [118] and EBV from Bioproject PRJNA518137 [119]. WGS generated during this study are available at the Sequence Read Archive (SRA) repository under accession number PRJNA1152337 and RNA-Seq datasets of this study have been deposited with the GEO data bank under accession number GSE274887.
