# Supplementary figures and images for: Transcriptomic characterisation of acute myeloid leukemia cell lines bearing the same t(9;11) driver mutation reveals different molecular signatures
Source: BMC Genomics. 2025 Mar 25;26:300. doi: 10.1186/s12864-025-11415-1 (PMC11938659; doi:10.1186/s12864-025-11415-1)

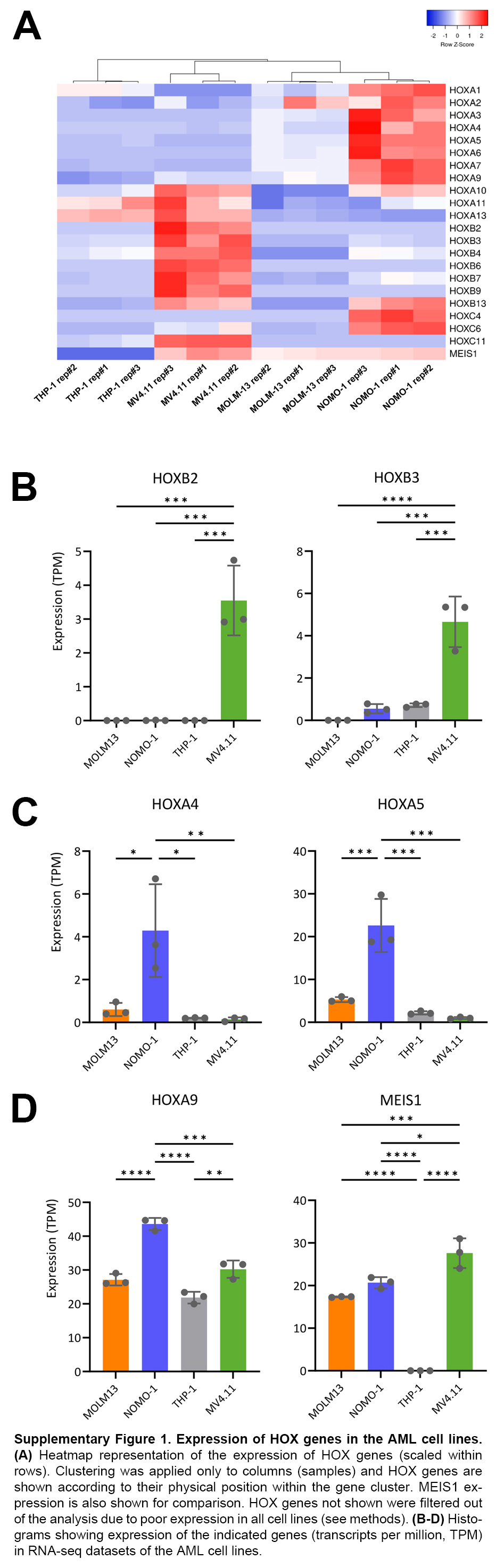

Supplement: Supplementary file 1 — Supplementary Material 1 [file 12864_2025_11415_MOESM1_ESM.jpg]

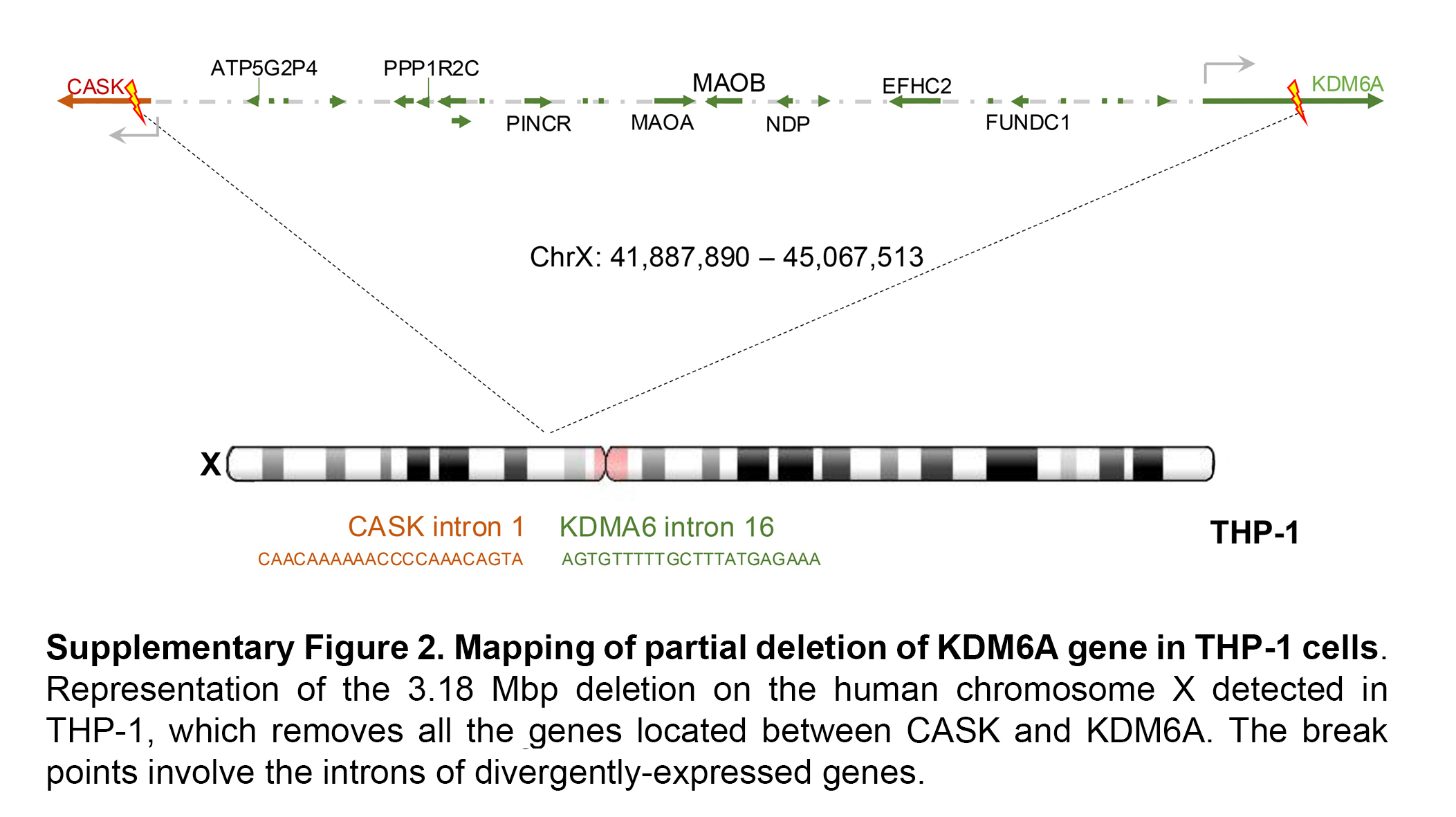

Supplement: Supplementary file 2 — Supplementary Material 2 [file 12864_2025_11415_MOESM2_ESM.jpg]

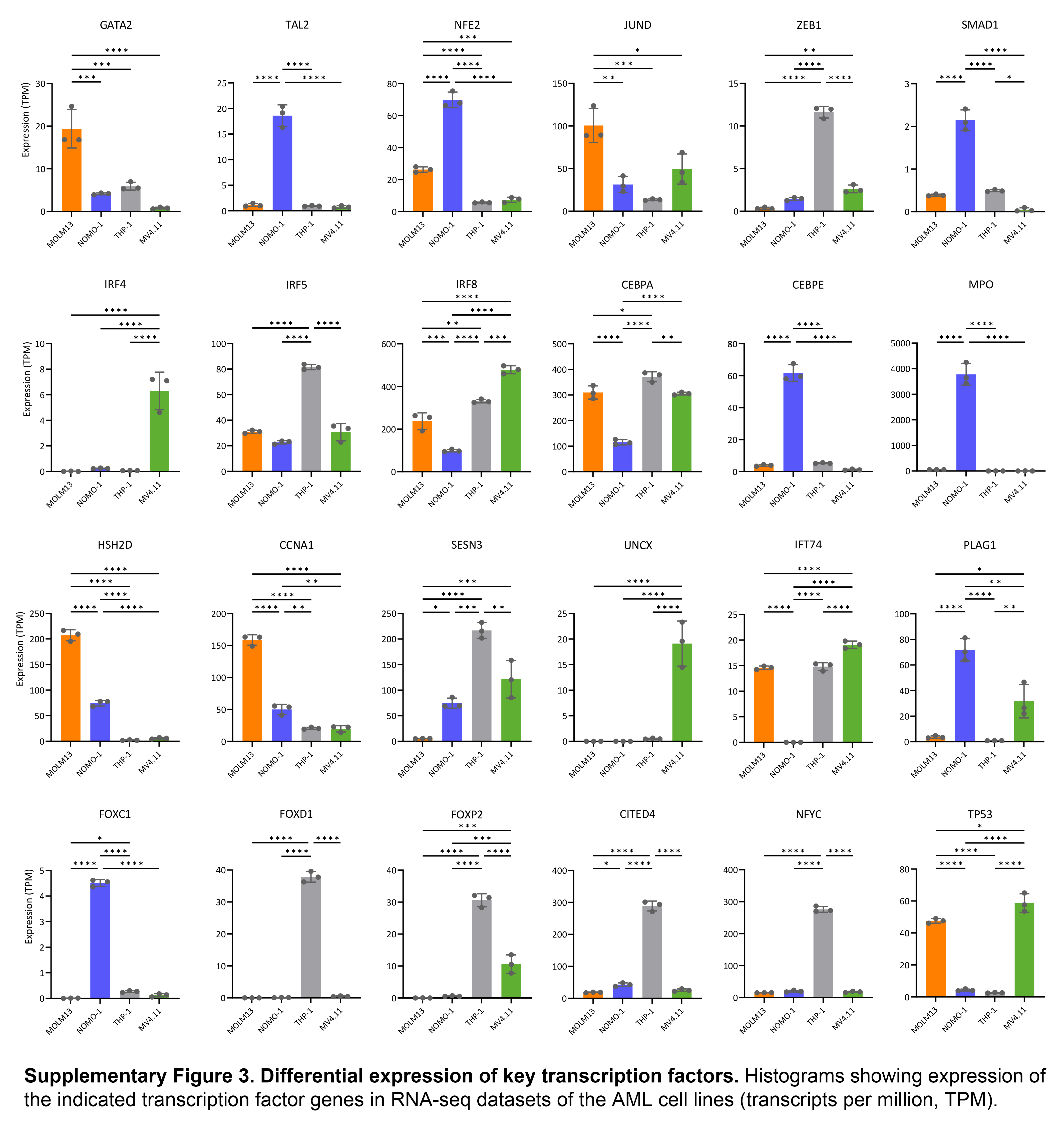

Supplement: Supplementary file 3 — Supplementary Material 3 [file 12864_2025_11415_MOESM3_ESM.jpg]

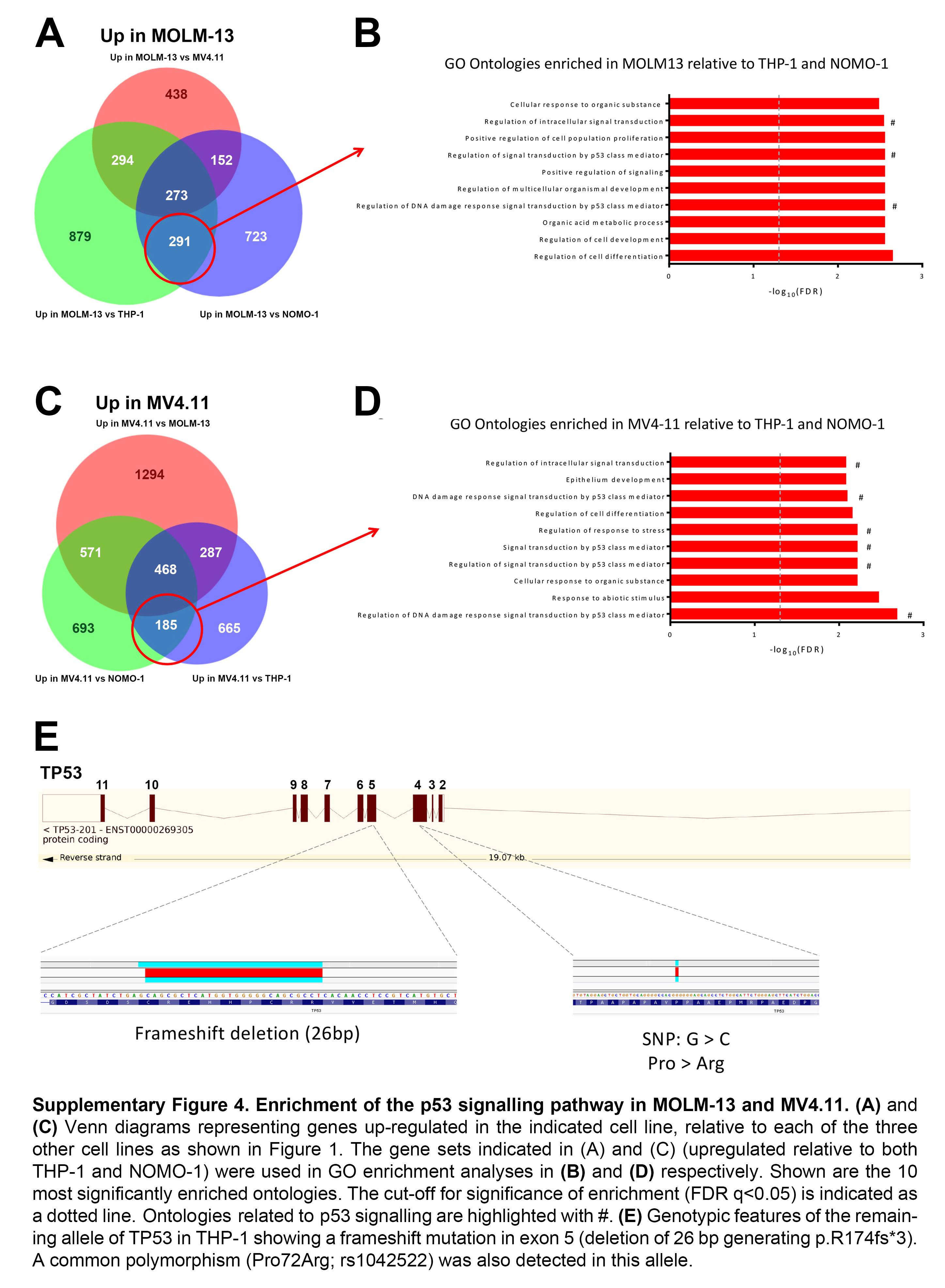

Supplement: Supplementary file 4 — Supplementary Material 4 [file 12864_2025_11415_MOESM4_ESM.jpg]

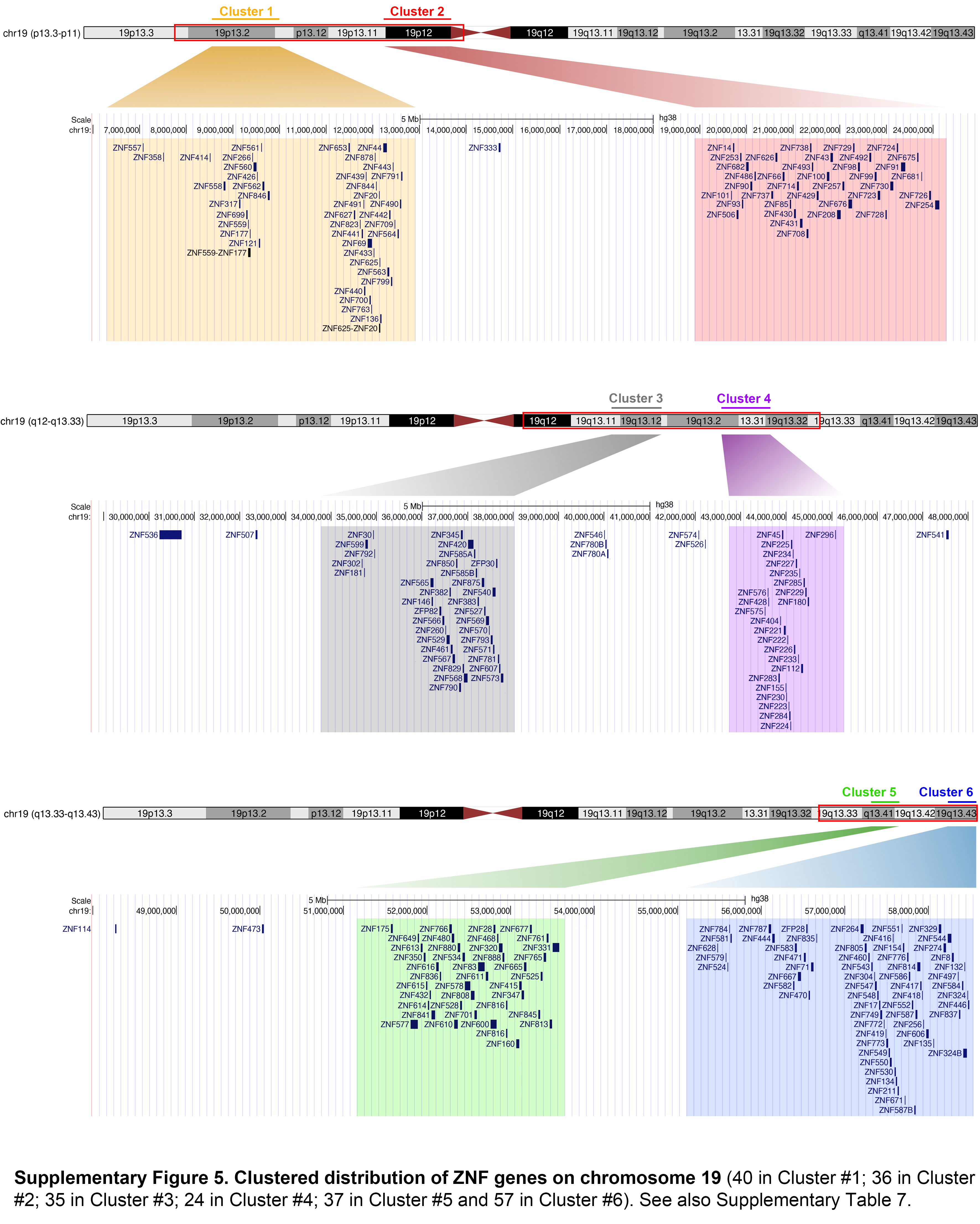

Supplement: Supplementary file 5 — Supplementary Material 5 [file 12864_2025_11415_MOESM5_ESM.jpg]

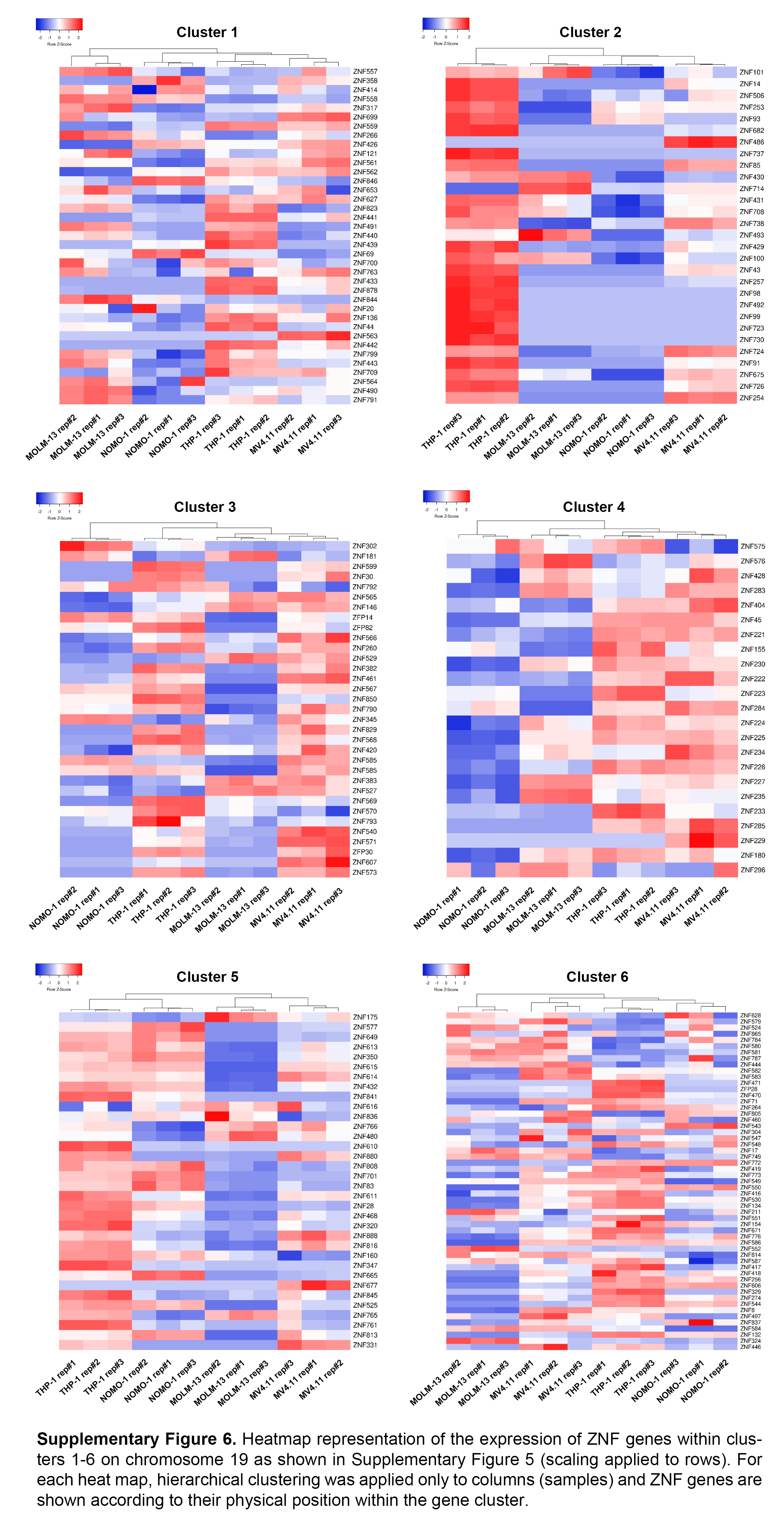

Supplement: Supplementary file 6 — Supplementary Material 6 [file 12864_2025_11415_MOESM6_ESM.jpg]
