# Supplementary material for: Transcriptomic characterisation of acute myeloid leukemia cell lines bearing the same t(9;11) driver mutation reveals different molecular signatures
Source: BMC Genomics. 2025 Mar 25;26:300. doi: 10.1186/s12864-025-11415-1 (PMC11938659; doi:10.1186/s12864-025-11415-1)
Supplement: Supplementary file 14 — Supplementary Material 14 [file 12864_2025_11415_MOESM14_ESM.docx]

| **Sample ID** | **Number of read pairs** | **Mapped reads %** | **Multimapped reads %** |
| --- | --- | --- | --- |
| **MOLM13 - rep1** | 120,815,492 | 93.50 | 5.90 |
| **MOLM13 - rep2** | 136,885,486 | 92.59 | 6.73 |
| **MOLM13 - rep3** | 116,543,027 | 91.69 | 5.61 |
| **MV4.11 - rep1** | 125,028,066 | 94.46 | 6.46 |
| **MV4.11 - rep2** | 114,331,209 | 93.58 | 6.25 |
| **MV4.11 - rep3** | 113,873,551 | 92.72 | 5.88 |
| **NOMO-1 - rep1** | 117,980,792 | 93.87 | 5.81 |
| **NOMO-1 - rep2** | 116,085,071 | 92.91 | 5.68 |
| **NOMO-1 - rep3** | 113,847,541 | 90.91 | 10.39 |
| **THP-1 - rep1** | 131,709,936 | 91.60 | 6.78 |
| **THP-1 - rep2** | 121,521,156 | 90.38 | 6.60 |
| **THP-1 - rep3** | 120,045,013 | 91.20 | 6.52 |

**Suppl Table 8.** Mapping statistics of the RNA-Seq experiments after adapter removal. Reads were mapped against the GRCh38 genome.
